# Systemic increase in IL-26 is associated with severe COVID-19 and comorbid obstructive lung disease

**DOI:** 10.3389/fimmu.2024.1434186

**Published:** 2024-10-04

**Authors:** Eduardo I. Cardenas, Josefina Robertson, Salvia Misaghian, Jermaine Brown, Mingyue Wang, Martin Stengelin, George Sigal, Jacob Wohlstadter, Magnus Gisslén, Anders Lindén

**Affiliations:** ^1^ Division of Lung and Airway Research, Institute of Environmental Medicine, and the Center for Molecular Medicine, Karolinska Institutet, Stockholm, Sweden; ^2^ Division of Ear, Nose and Throat Diseases, Department of Clinical Science, Intervention and Technology, Karolinska Institutet, Stockholm, Sweden; ^3^ Department of Infectious Diseases, Institute of Biomedicine, Sahlgrenska Academy, University of Gothenburg, Gothenburg, Sweden; ^4^ Department of Infectious Diseases, Region Västra Götaland, Sahlgrenska University Hospital, Gothenburg, Sweden; ^5^ Meso Scale Diagnostics, LLC., Rockville, MD, United States; ^6^ Public Health Agency of Sweden, Stockholm, Sweden; ^7^ Karolinska Severe COPD Center, Department of Respiratory Medicine and Allergy, Karolinska University Hospital, Stockholm, Sweden

**Keywords:** IL-26, COVID-19, SARS-CoV-2, asthma, COPD

## Abstract

**Background:**

IL-26 is a key mediator of pulmonary host defense given its abundant expression in human airways and its established antibacterial properties. Moreover, recent studies indicate that IL-26 can also inhibit viral replication. Along these lines, we have previously reported an increase in the plasma concentration of IL-26 among patients with acute COVID-19 that is linked to harmful hyperinflammation. Nevertheless, it is still unclear whether this systemic increase in IL-26 relates to disease severity, sex, comorbidities, viral load, or the innate immune response in acute COVID-19.

**Methods:**

IL-26 was quantified using ELISA in plasma samples from a large cohort of well-characterized patients with acute COVID-19 (n=178) and healthy controls (n=30). The plasma concentrations of SARS-CoV-2 nucleocapsid and spike protein, as well as those of IFN-α2a, IFN-β, and IFN-γ, were determined using electrochemiluminescence immunoassay. The concentration of double-stranded DNA was determined using fluorometry.

**Results:**

The plasma concentration of IL-26 was increased in patients with severe/critical COVID-19, particularly among males and patients with comorbid obstructive lung disease. Moreover, the concentration of IL-26 displayed positive correlations with length of hospital stay, as well as with systemic markers of viral load, antiviral immunity, and extracellular DNA.

**Conclusions:**

Systemic IL-26 is involved in severe COVID-19, especially in males and patients with comorbid obstructive lung disease. These findings argue that systemic IL-26 has pathogenic and antiviral relevance, as well as biomarker potential.

## 1 Introduction

Interleukin (IL)-26 is abundantly expressed in human airways (1), where it is thought to play a major role in host defense due to its potent anti-bacterial (2, 3) and neutrophil-mobilizing properties (4–6). Moreover, an expanding literature suggests that IL-26 participates in anti-viral immune responses. In fact, IL-26 was first identified in virally-infected T-lymphocytes (7), and IL-26 is often co-expressed with interferon (IFN)-γ in different lymphocyte subsets (8–10). Furthermore, IL-26 inhibits the replication of the Hepatitis C virus (HCV) in hepatocytes via direct binding to RNA replication intermediates (11). Given that both HCV and the severe acute respiratory syndrome coronavirus 2 (SARS-CoV-2) contain positive-sense single-stranded RNA genomes, it is plausible that IL-26 may play a comparable role in modulating the immune response against SARS-CoV-2. In line with this, we have previously demonstrated that stimulation of Toll-like receptors (TLRs) involved in the innate recognition of SARS-CoV-2 (i.e., TLR2, -3, -4, -7 and -8) triggers the release of IL-26 in different structural and immune cells isolated from human airways (3, 4, 12–17). In addition, we recently published evidence that systemic IL-26 is markedly increased in patients with acute coronavirus disease 2019 (COVID-19), and that this increase associates with hyperinflammation and tissue damage (18). Taken together, these findings highlight the potential of systemic IL-26 as a target for therapy in COVID-19. However, to evaluate this potential, we need to improve the understanding of how systemic IL-26 relates to disease severity and respiratory comorbidity.

In the present study, we utilized a large cohort of patients with acute COVID-19 and healthy controls to characterize how systemic IL-26 relates to disease severity, sex and comorbidities. We also addressed the corresponding associations with markers of viral load, the antiviral innate immune response, and a marker of NET formation and/or tissue damage. In doing so, we obtained evidence that systemic IL-26 associates with all the referred aspects of COVID-19, including respiratory comorbidity, thereby reinforcing the potential of systemic IL-26 as a target with clinical utility.

## 2 Methods

### 2.1 Patient material

All COVID-19 patients admitted between February 2020 and January 2021 at the Department of Infectious Diseases, Sahlgrenska University Hospital (Gothenburg, Sweden) were eligible for inclusion. In parallel, a healthy control group was recruited for comparison **Table 1**. SARS-CoV-2 infection was confirmed via reverse transcriptase polymerase chain reaction (RT-PCR) from nasopharyngeal and throat aspirates, and all healthy controls had a negative test. Blood was collected at a single time-point and written informed consent was provided by all participants before sample collection. All procedures and handling of patient information were conducted in accordance with the World Medical Associations recommendations (the Declaration of Helsinki) and the ethical permit approved by the Swedish Ethical Review Authority (Diary No. 2020-01771). A subset of patients included in this study have been described previously (19–22).

**Table 1:** Characteristics of study participants.

	COVID-19				Healthy (n=30)
	Mild (n=13)	Moderate (n=96)	Severe/Critical (n=69)	All (n=178)	
**Age (years)**	49 (32–73)	53 (24–87)	54 (23–82)	53 (23–87)	58.5 (26–82)
**Females/Males (n)**	6/7	31/65	16/53	53/125	10/20
**Time between onset of symptoms and sample collection (days)**	8 (1–31)	10 (2–22)	9 (3–19)	9 (1–31)	N/A
**Length of hospital stay (days)**	0 (0–6)	5 (2–18)	15 (4–102)	7 (0–102)	N/A
**Corticosteroid use (n, %)**	0 (0%)	39 (41%)	33 (48%)	72 (40%)	N/A
**Comorbidities (n, %)**	9 (69%)	59 (61%)	47 (68%)	115 (65%)	N/A
Hypertension	2 (15%)	29 (30%)	23 (33%)	54 (30%)	N/A
Type 2 Diabetes	1 (8%)	14 (15%)	13 (19%)	28 (16%)	N/A
Obstructive Lung Disease	2 (15%)	13 (14%)	12 (17%)	27 (15%)	N/A

All data are presented as median (range) unless indicated otherwise. Obstructive Lung Disease encompasses Asthma and COPD. N/A, Not applicable.

### 2.2 Quantification of cytokines, SARS-CoV-2 antigens, and dsDNA in human plasma

The systemic protein concentration of IL-26 was assessed in plasma samples (1:2 dilution) via ELISA (15.6–4000 pg/ml detection range; Cusabio). The plasma concentrations of SARS-CoV-2 nucleocapsid and spike proteins (1:2 dilution), as well as the protein concentrations of IFN-α2a, IFN-β, and IFN-γ (all in undiluted samples), were determined using commercially available S-PLEX^®^ kits (Meso Scale Discovery^®^) via electrochemiluminescence immunoassay (23). The detection ranges of these kits were as follows: 0.16 – 1100 pg/ml for SARS-CoV-2 nucleocapsid protein, 0.28 – 376 pg/ml for SARS-CoV-2 spike protein, 0.0053 – 33 pg/ml for IFN-α2a, 0.0065 – 33 pg/ml for IFN-β, and 0.0048 – 29 pg/ml for IFN-γ. The concentration of double-stranded DNA (dsDNA) in undiluted plasma samples was determined in a Qubit 3.0 Fluorometer using the Qubit 1X dsDNA BR Assay kit (20 – 20000 ng/ml detection range; both from ThermoFisher). Of note, the concentrations of viral antigens, interferons, and dsDNA could only be measured in 177, 149, and 176 samples, respectively, due to sample depletion.

### 2.3 Statistical analyses

Multiple comparisons were conducted via non-parametric Kruskal-Wallis test followed by Dunn’s *post hoc* test to compare each group against the others. Pairwise comparisons were performed by unpaired non-parametric Mann-Whitney test. Associations between two continuous variables were assessed by Spearman’s rank correlation test. Non-parametric methods were chosen given that all comparisons included at least one group that failed the D’Agostino & Pearson normality test. Pairwise comparisons of categorical data were conducted via chi-square (*χ2*) test. For the multivariable logistic regression analysis, all variables were transformed into categorical data as described in **Table 2**. For the transformation into categorical data of our results on viral antigens, IFNs, and dsDNA, we established whether each individual value was above or below the median of its corresponding COVID-19 severity group. The receiver operating characteristic (ROC) curve and its associated area under the curve were calculated to assess the prediction capacity of the generated logistic regression model.

**Table 2:** Multivariable logistic regression analysis.

Variable	Reference	O.R.	95% C.I. of O.R.	p-value	V.I.F.
**Severe/Critical COVID-19 (Yes/No)**	No	3.420	1.587–7.745	0.0022	1.032
**Sex (Male/Female)**	Female	2.552	1.038–6.658	0.0466	1.120
**>9 days between onset of symptoms and sample collection (Yes/No)**	Yes	0.4683	0.196–1.068	0.0776	1.178
**Obstructive Lung Disease (Yes/No)**	No	4.270	1.472–13.54	0.0097	1.098
**SARS-CoV-2 Nucleocapsid** above median (Yes/No)*	No	1.332	0.474–3.795	0.5866	2.055
**SARS-CoV-2 Spike protein** above median (Yes/No)*	No	1.208	0.469–3.073	0.6910	1.642
IFN-α2a above median (Yes/No)*	No	1.245	0.475–3.261	0.6537	1.691
IFN-β above median (Yes/No)*	No	2.061	0.875–4.943	0.0994	1.367
IFN-γ above median (Yes/No)*	No	2.565	1.079–6.298	0.0351	1.387
dsDNA above median (Yes/No)*	No	2.873	1.289–6.667	0.0113	1.181
**Intercept**	N/A	0.043	0.011–0.147	<0.0001	N/A

O.R., odds ratio; C.I., confidence interval; V.I.F., Variance Inflation Factor; N/A, Not applicable. *See methods for an explanation on medians used.

## 3 Results

### 3.1 Patient characteristics

One hundred and seventy-eight patients with COVID-19 and 30 age- and sex-matched healthy controls were enrolled in the study. The main characteristics of all study participants are summarized in **Table 1** (see also Methods for more information). Disease severity was classified according to the COVID-19 Treatment Guidelines Panel of the National Institutes of Health (24). Mild disease included patients not requiring oxygen nor in-patient hospital care; moderate disease included hospitalized patients receiving oxygen therapy by mask or nasal cannula; and severe/critical disease included hospitalized patients in need of high flow nasal oxygen (HFNO) or mechanical ventilator. More than half of all patients with COVID-19 had at least one comorbidity regardless of disease severity, with hypertension, type 2 diabetes, and obstructive lung disease being the most frequent. In general, patients with severe/critical COVID-19 had prolonged length of hospital stay and a higher incidence of comorbidities.

### 3.2 Increased plasma concentrations of IL-26 associate with severe COVID-19, male sex and comorbid obstructive lung disease

**Figure 1:**
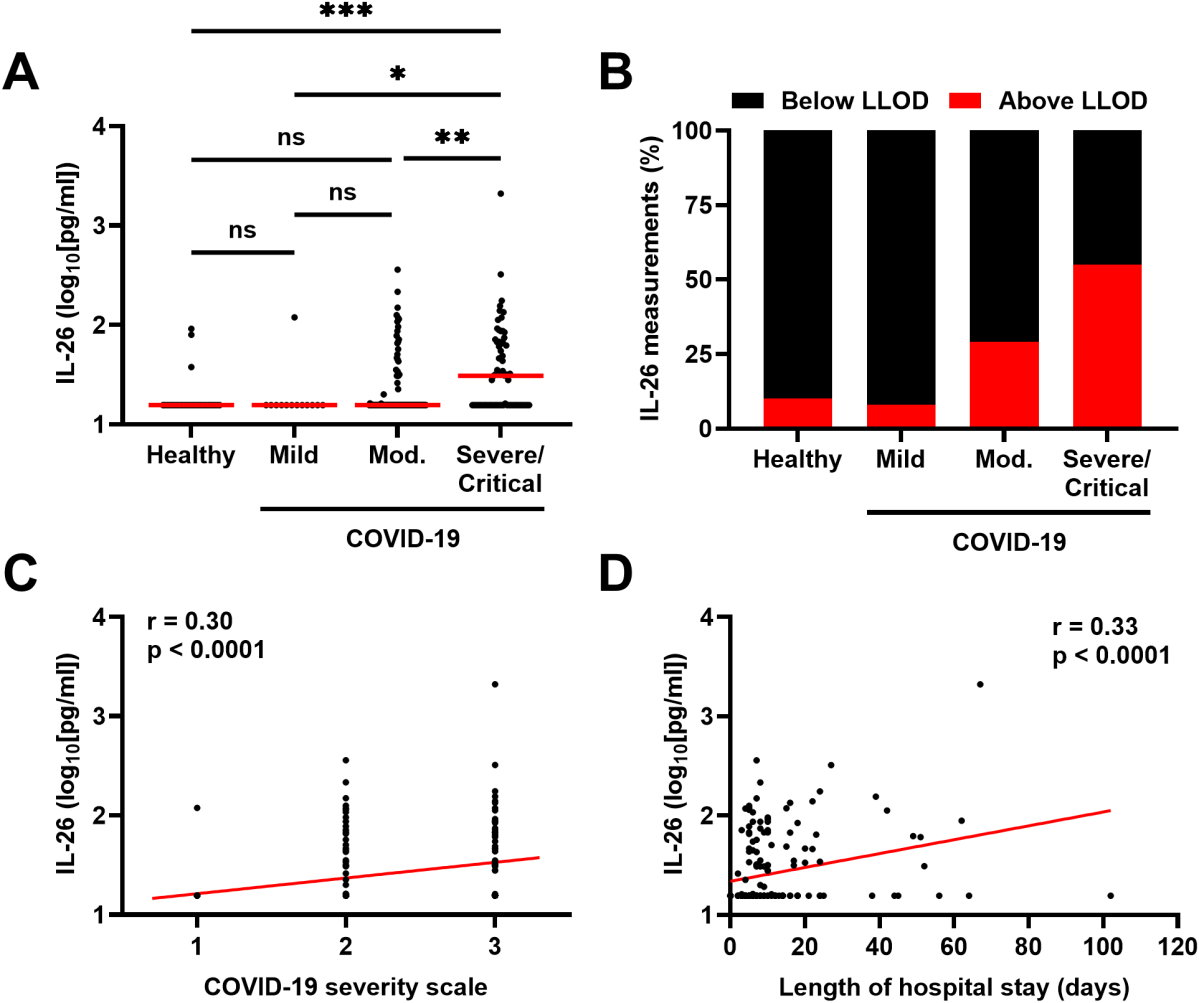
Relationship between the plasma concentration of IL-26 and severity of COVID-19. **(A)** Comparison of the plasma concentrations of IL-26 among healthy controls (n=30) and patients with mild (n=13), moderate (Mod.; n=96), or severe/critical (n=69) COVID-19 by Kruskal-Wallis test followed by Dunn’s *post hoc* test. Red horizontal lines represent the median. **(B)** Percentage of samples in which the plasma concentration of IL-26 fell below (black) or above (red) the lower limit of detection (LLOD) in each study group. Spearman correlation analyses of the plasma concentration of IL-26 with **(C)** the COVID-19 severity scale (1=mild, 2=moderate, and 3=severe/critical) and **(D)** the length of hospital stay of each study participant (n=178). *p < 0.05, **p < 0.005, ***p < 0.0005, ns, not significant.

The concentrations of IL-26 in plasma were higher in patients with severe/critical COVID-19 compared to those with mild and moderate disease, as well as to healthy controls (**Figure 1A**). Moreover, the number of plasma samples with a concentration of IL-26 above the lower limit of detection (LLOD) increased from 10% in samples from healthy controls and patients with mild COVID-19, to 30 and 55% in samples from patients with moderate and severe/critical COVID-19, respectively (**Figure 1B**). In addition, the concentration of IL-26 in plasma displayed positive correlations with disease severity (**Figure 1C**) and length of hospital stay (**Figure 1D**) in patients with COVID-19.

**Figure 2:**
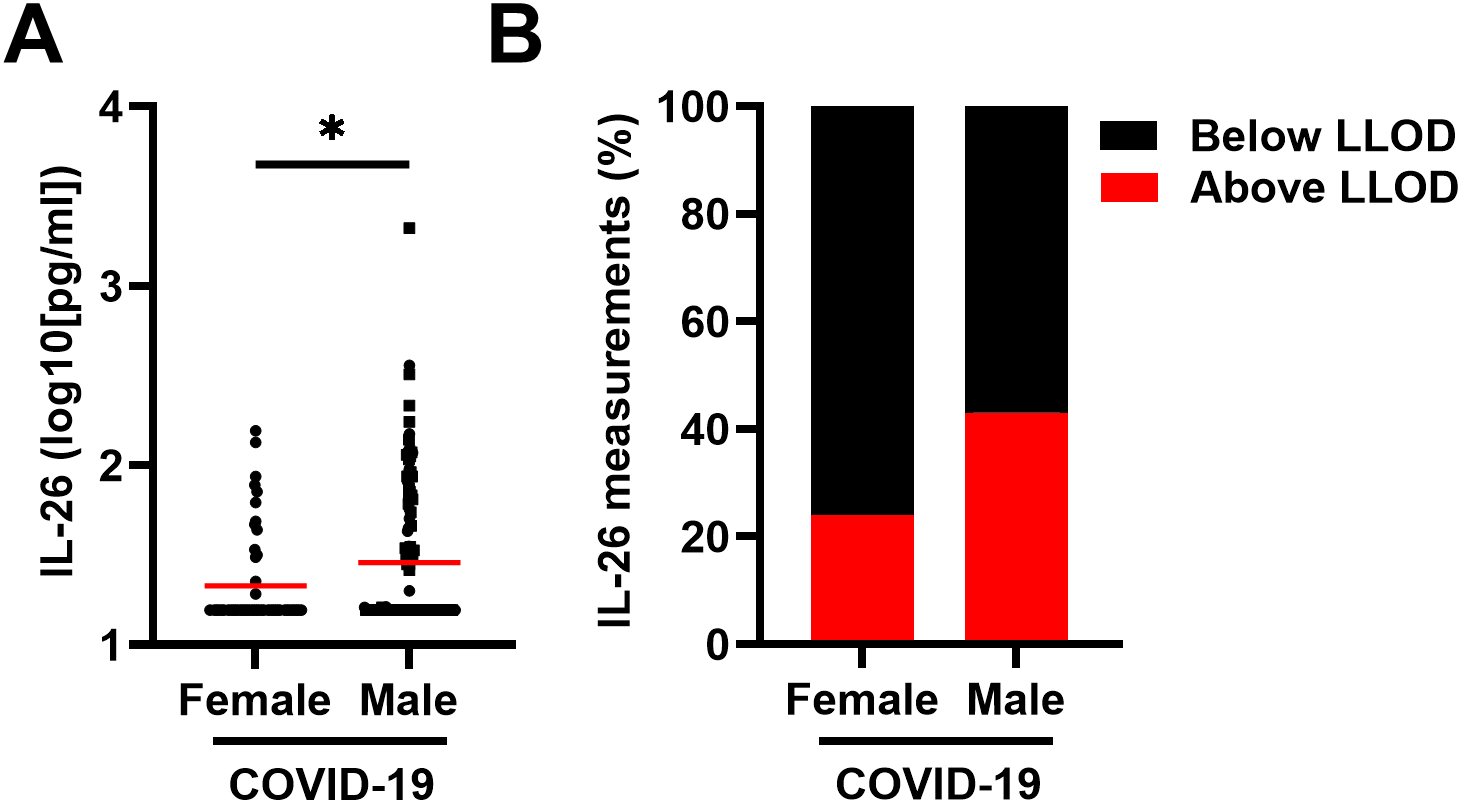
Relationship between the plasma concentration of IL-26 and sex in patients with COVID-19. **(A)** Comparison of the plasma concentrations of IL-26 between female (n=53) and male (n=125) patients with COVID-19 by unpaired Mann-Whitney test. Red horizontal lines represent the median. **(B)** Percentage of samples in which the plasma concentration of IL-26 fell below (black) or above (red) the lower limit of detection (LLOD) in each study group. *p < 0.05.

Notably, the concentrations of IL-26 in plasma were higher in males than in females with COVID-19 (**Figure 2A**) and the number of plasma samples with a concentration of IL-26 above the LLOD were higher (*χ2* test: p = 0.0187) in males than in females with COVID-19 (**Figure 2B**).

**Figure 3:**
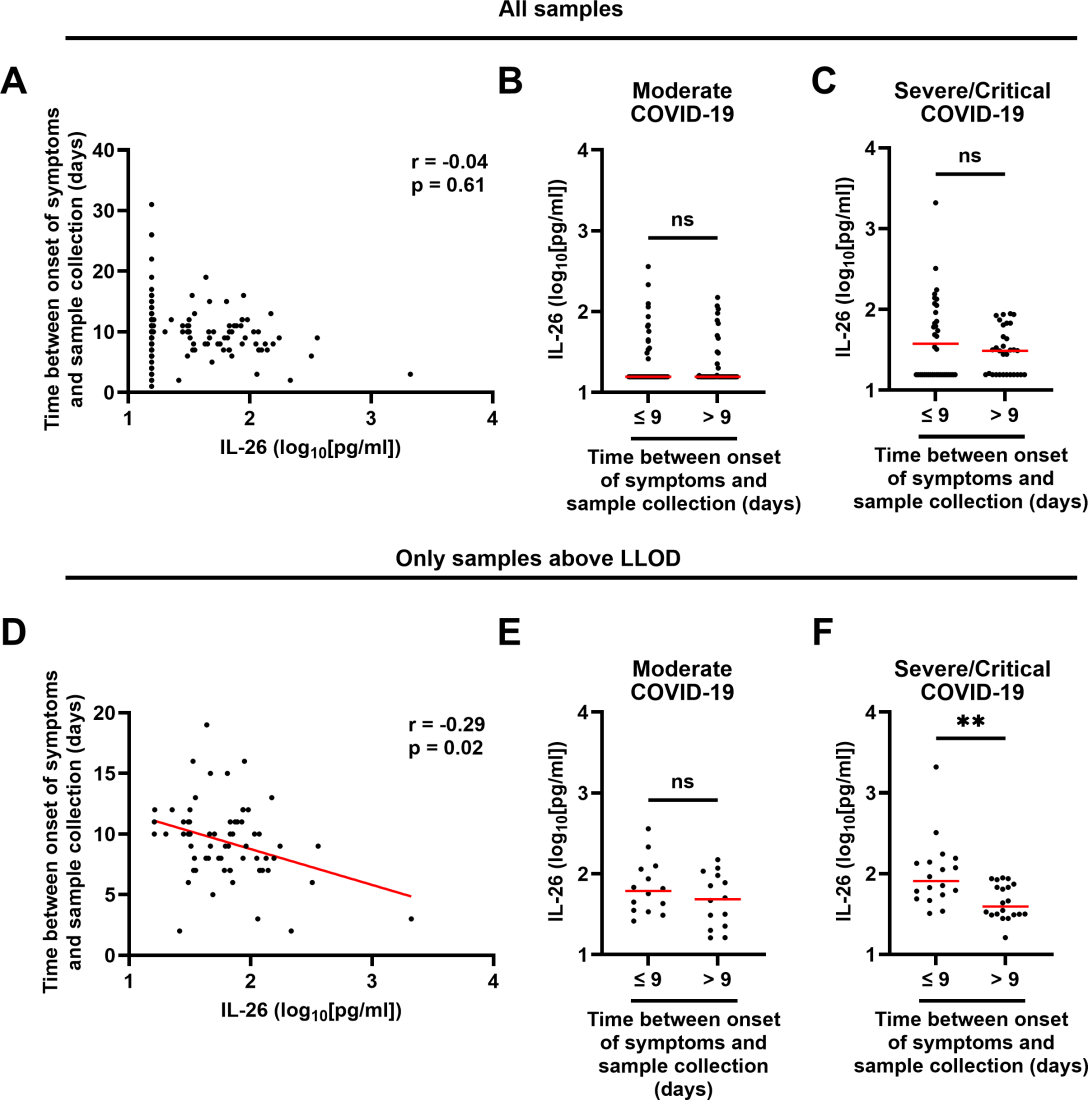
Relationship between the plasma concentration of IL-26 and the time between onset of symptoms and sample collection in patients with COVID-19. **(A, D)** Spearman correlation analyses of the plasma concentrations of IL-26 with the time between onset of symptoms and sample collection in **(A)** all samples (n=178) and **(D)** only samples with an IL-26 concentration above the lower limit of detection (LLOD; n=67). Comparisons of the plasma concentrations of IL-26 between samples collected before or after the median time since onset of symptoms in patients with **(B, E)** moderate or **(C, F)** severe/critical COVID-19 including **(B, C)** all samples, or **(E, F)** only samples with an IL-26 concentration above the LLOD, by unpaired Mann-Whitney test. Red horizontal lines represent the median. **p < 0.005, ns, not significant.

The median time between onset of symptoms and sample collection was similar across groups (9 days; **Table 1**) and did not display a statistically significant correlation with the concentration of IL-26 when all samples were included (**Figure 3A**). Moreover, comparison of the IL-26 concentrations in samples collected before or after the median time from onset of symptoms revealed no statistically significant differences when all samples were included (**Figures 3B, C**). Furthermore, the number of samples with a concentration of IL-26 above the LLOD was similar (*χ2* test: p = 0.5714; **Supplementary Figure 1**) in samples collected before or after the median time from onset of symptoms. However, the concentration of IL-26 displayed a negative correlation with the time between onset of symptoms and sample collection when all IL-26 measurements below the lower limit of detection were excluded (**Figure 3D**). In addition, exclusion of samples with an IL-26 concentration below the LLOD revealed that samples collected within 9 days from onset of symptoms had a higher IL-26 concentration than those collected afterwards in patients with severe/critical COVID-19 exclusively (**Figures 3E, F**). Of note, samples from patients with mild COVID-19 were excluded from this analysis given that only 1 of them had an IL-26 concentration above the lower limit of detection (**Figure 1A**).

**Figure 4:**
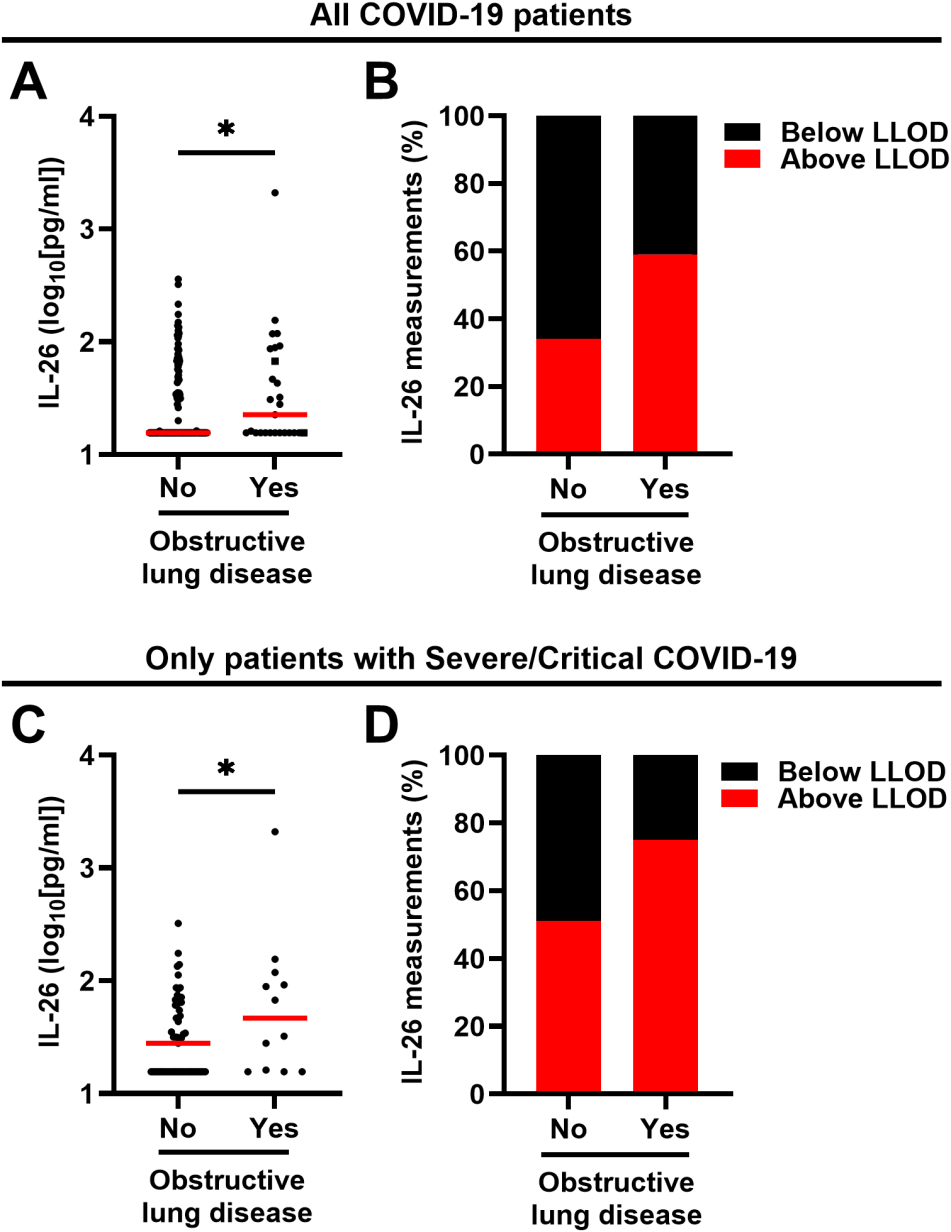
Relationship between the plasma concentration of IL-26 and the respiratory comorbidity obstructive lung disease in patients with COVID-19. **(A, C)** Comparisons of the plasma concentrations of IL-26 between patients with and without comorbid obstructive lung disease including **(A)** all patients with COVID-19 (n=178) or **(C)** only those with severe/critical COVID-19 (n=69) by **(A)** two-tailed or **(C)** one-tailed unpaired Mann-Whitney test. Red horizontal lines represent the median. **(B, D)** Percentage of samples in which the plasma concentration of IL-26 fell below (black) or above (red) the lower limit of detection (LLOD) in each study group including **(B)** all patients with COVID-19 (n=178) or **(C)** only those with severe/critical COVID-19. *p < 0.05, ns, not significant.

We also investigated whether common comorbidities had an impact on the plasma concentration of IL-26 among all patients with COVID-19 and found that patients with a prior diagnosis of obstructive lung disease (asthma: n=22; COPD: n=4; asthma and COPD: n=1) displayed a higher plasma concentration of IL-26 (**Figure 4A**) and a clearly higher (*χ2* test: p = 0.0370) number of samples with a concentration of IL-26 above the lower limit of detection (**Figure 4B**). In contrast, no matching differences were observed in patients with comorbid hypertension or diabetes type 2 (**Supplementary Figure 2**). A similar increase in IL-26 concentrations and number of samples above the LLOD (*χ2* test: p = 0.0634) was observed for obstructive lung disease in patients with severe/critical COVID-19 (**Figures 4C, D**). Importantly, patients with COVID-19 who took corticosteroids as part of their treatment had similar plasma concentrations of IL-26 and a similar number of measurements above the LLOD (*χ2* test: p = 0.3608) than those who did not receive corticosteroids (**Supplementary Figure 3**).

### 3.3 The plasma concentrations of IL-26 correlate with markers of viral load in patients with COVID-19

**Figure 5:**
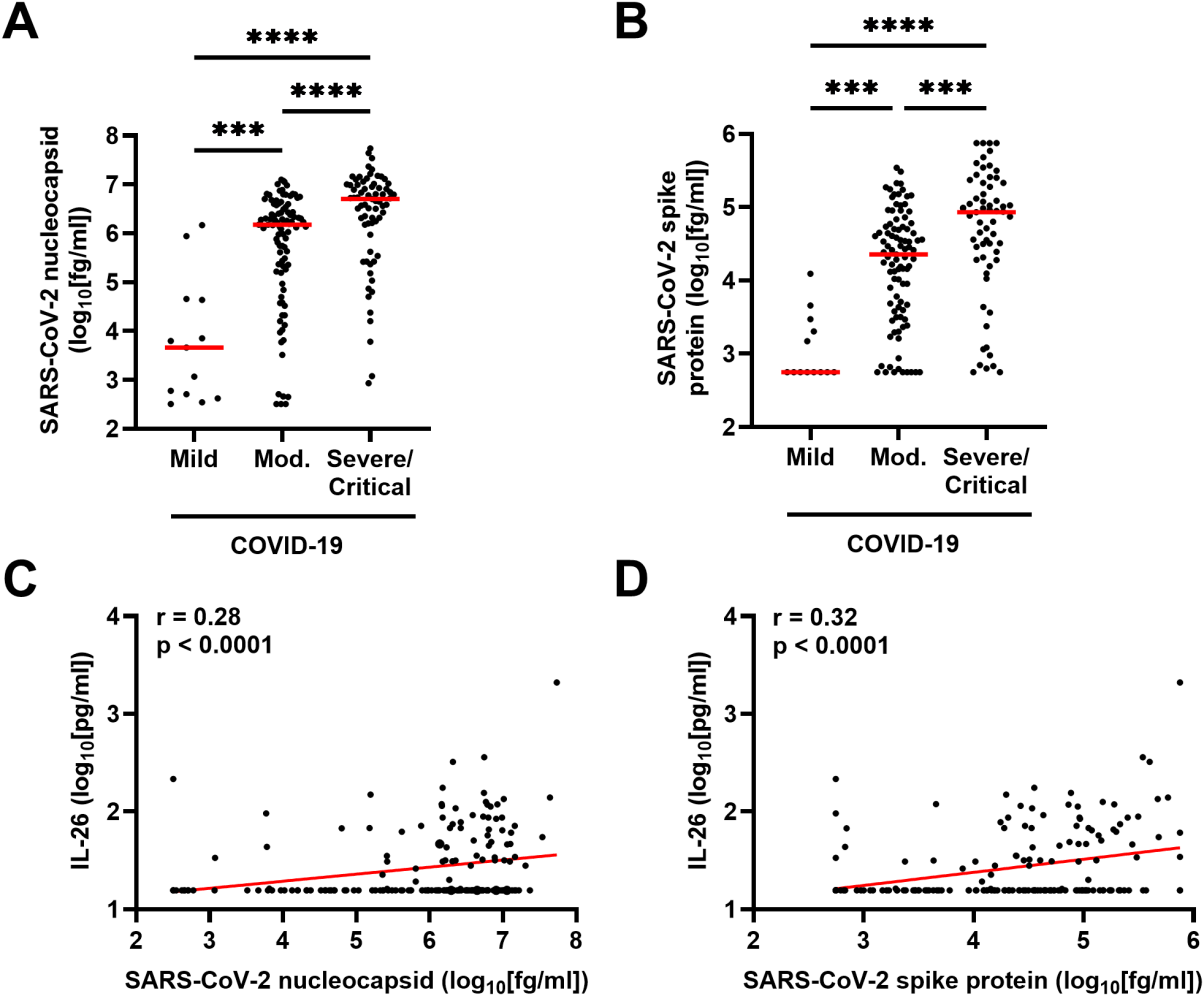
Relationship between plasma concentration of IL-26 and markers of viral load in patients with COVID-19. Comparison of the plasma concentrations of SARS-CoV-2 **(A)** nucleocapsid and **(B)** spike protein among patients with mild (n=13), moderate (n=95), or severe/critical (n=69) COVID-19 by Kruskal-Wallis test followed by Dunn’s *post hoc* test. Spearman correlation analyses of the plasma concentration of IL-26 with those of **(C)** SARS-CoV-2 nucleocapsid and **(D)** SARS-CoV-2 spike protein (n=177). Red horizontal lines represent the median. ***p < 0.0005, ****p < 0.0001.

To determine whether a higher viral load was linked to an increase in IL-26, we first quantified the plasma concentrations of two SARS-CoV-2 antigens in patients with COVID-19. As expected, higher concentrations of SARS-CoV-2 nucleocapsid and spike protein corresponded with more severe disease (**Figures 5A, B**). Moreover, the plasma concentration of IL-26 displayed positive correlations with both markers of viral load in patients with COVID-19 (**Figures 5C, D**).

### 3.4 The plasma concentrations of IL-26 associate with the magnitude of the antiviral innate immune response in patients with COVID-19

**Figure 6:**
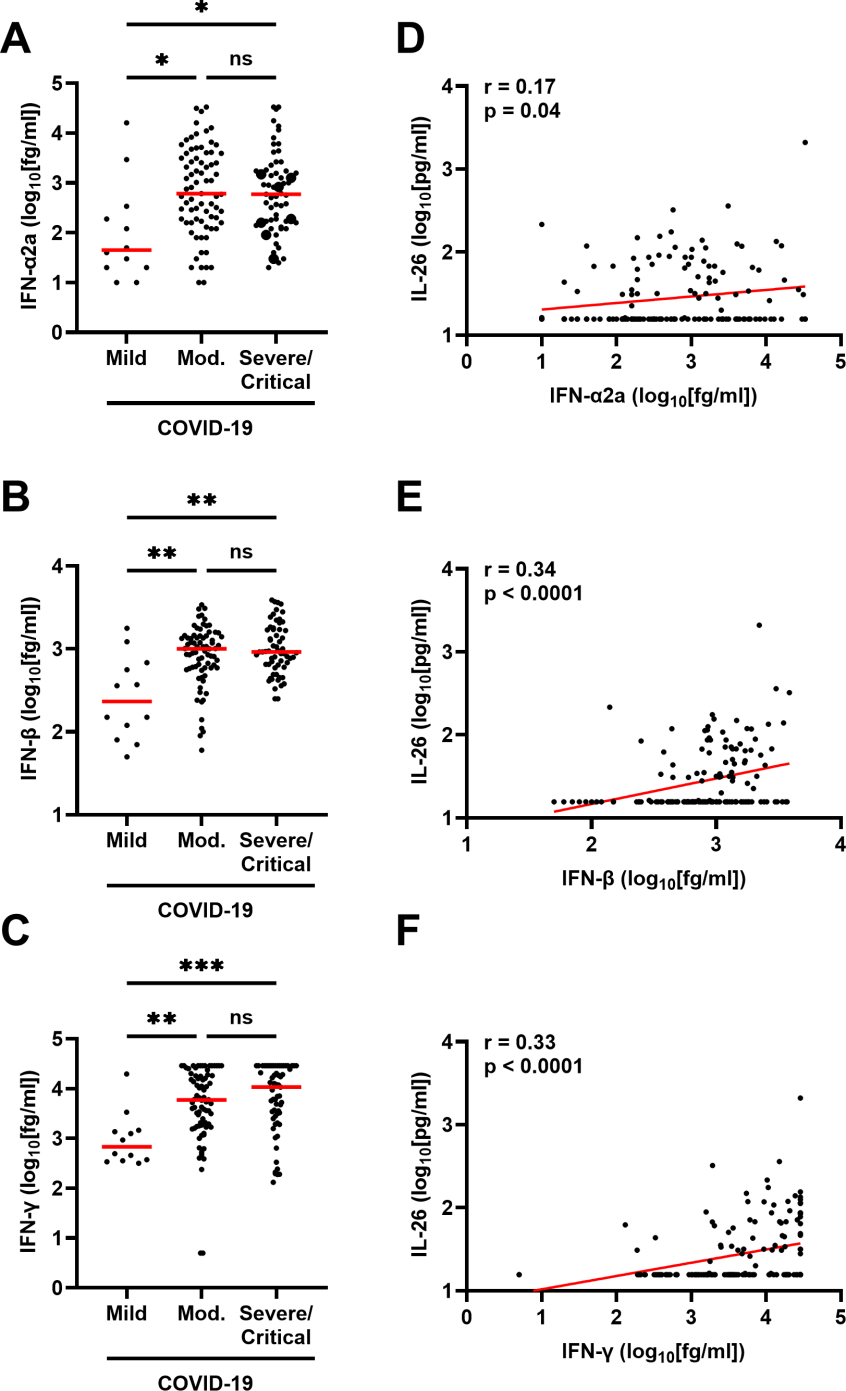
Relationship between plasma concentration of IL-26 and production of interferons in patients with COVID-19. Comparison of the plasma concentrations of **(A)** IFN-α2a, **(B)** IFN-β, and **(C)** IFN-γ among patients with mild (n=12), moderate (n=76), or severe/critical (n=61) COVID-19 by Kruskal-Wallis test followed by Dunn’s *post hoc* test. Spearman correlation analyses of the plasma concentration of IL-26 with those of **(D)** IFN-α2a, **(E)** IFN-β, and **(F)** IFN-γ (n=149). Red horizontal lines represent the median. *p < 0.05, **p < 0.005, ***p< 0.0005, ns, not significant.

We also assessed whether the increase in plasma IL-26 associates with an increase in interferons—key markers of the antiviral innate immune response. Notably, the plasma concentrations of IFN-α2a, IFN-β, and IFN-γ were increased in patients with moderate or severe/critical COVID-19 compared to those with mild disease (**Figures 6A–C**). Moreover, the concentrations of these IFNs displayed positive correlations with that of IL-26 (**Figures 6D–F**).

### 3.5 The plasma concentrations of IL-26 correlate with that of extracellular dsDNA

**Figure 7:**
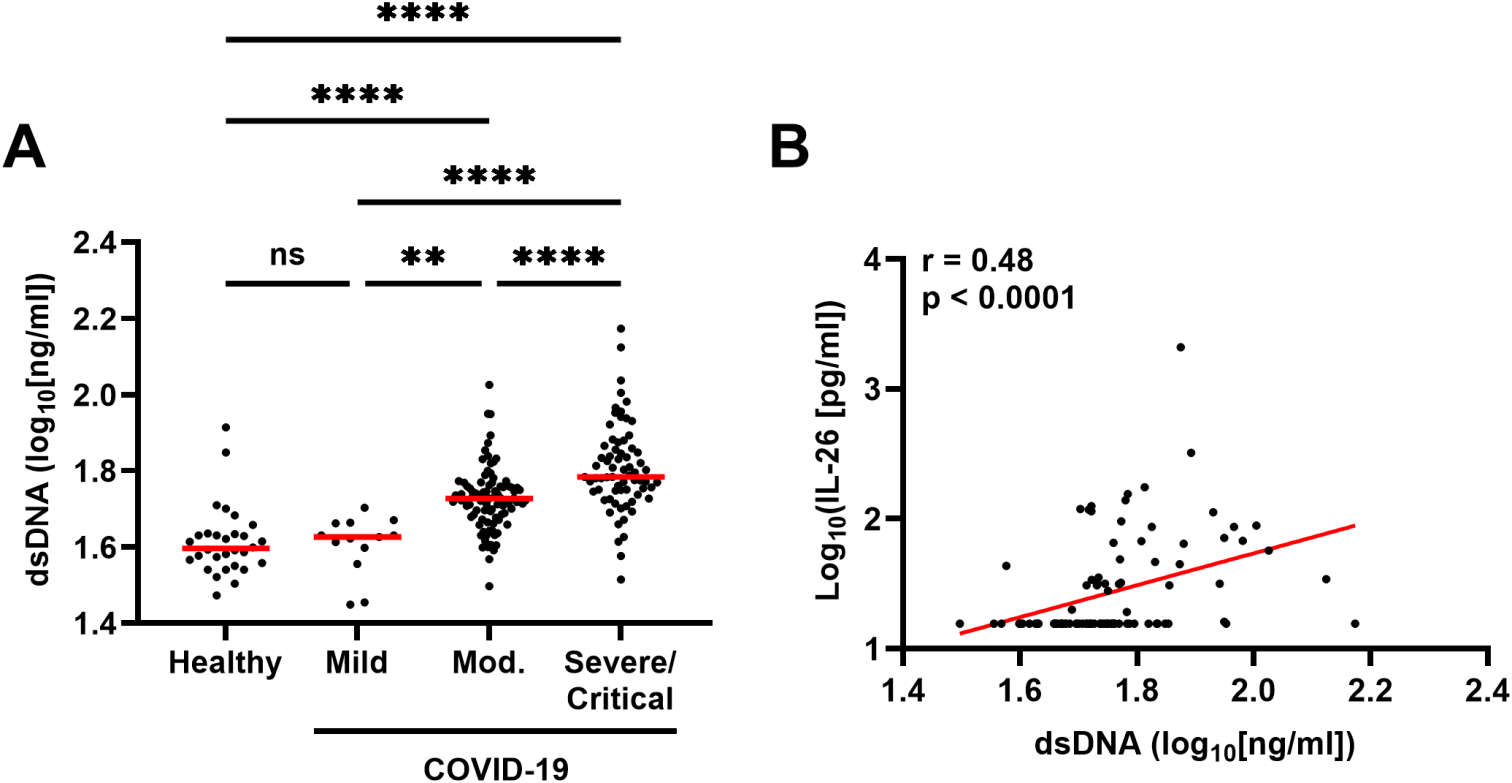
Relationship between the plasma concentration of IL-26 and double-stranded DNA (dsDNA) production in patients with COVID-19. **(A)** Comparison of the plasma concentrations of dsDNA among healthy controls (n=30) and patients with mild (n=13), moderate (n=94), or severe/critical (n=69) COVID-19 by Kruskal-Wallis test followed by Dunn’s *post hoc* test. **(B)** Spearman correlation analysis of the plasma concentration of IL-26 with that of dsDNA (n=176). Red horizontal lines represent the median. **p < 0.005, ****p < 0.0001, ns, not significant.

A higher concentration of dsDNA in plasma, which suggests increased NET production and/or tissue damage, was associated with more severe COVID-19 (**Figure 7A**) and a higher concentration of IL-26 (**Figure 7B**).

### 3.6 Multivariable logistic regression analysis

Finally, we performed a multivariable logistic regression analysis (**Table 2**) to determine whether each of the variables mentioned above associated with having a plasma concentration of IL-26 above the LLOD or not (Yes/No binary). We found that patients with severe/critical COVID-19 and males were 3.420 and 2.552 times more likely to have an IL-26 measurement (ELISA) above the LLOD than patients with mild/moderate COVID-19 and females, respectively. Notably, we found that patients with comorbid obstructive lung disease were 4.270 times more likely to have an IL-26 measurement above the LLOD than patients without this comorbidity. In addition, we found that patients with high plasma concentrations of IFN-γ and dsDNA (above their corresponding medians) were 2.107 and 2.532 times more likely to have an IL-26 measurement above the LLOD, respectively. On the other hand, time between onset of symptoms and sample collection, and the plasma concentrations of viral antigens, IFN-α2a or IFN-β were not associated with having IL-26 measurements above the LLOD. Importantly, all variables had a variance inflation factor (VIF) close to 1, which indicates low multicollinearity. Moreover, we calculated a ROC curve for this analysis (**Supplementary Figure 4**), and its associated area under the curve was 0.7881, which indicates that these variables had a good capacity to predict whether an IL-26 measurement would fall above the LLOD or not.

## 4 Discussion

In essence, the results of the current study substantially expand the evidence that IL-26 participates in the immune response against SARS-CoV-2, as well as in the hyperinflammation that characterizes severe cases of COVID-19, in several different ways.

First, and most important, we show that the increase in the plasma concentrations of IL-26 is most pronounced in patients with severe/critical disease and that it displays a positive correlation with length of hospital stay. This is in contrast with a previous study by a different group which reported no differences in the serum concentration of IL-26 among patients with mild, moderate, and severe COVID-19 (25). Nevertheless, we think that the limited size (n = 27) of the study by Caterino M. et al., and the fact that it only included 6 study participants with severe disease, made it difficult to identify statistical differences among sub-groups of varying disease severity. For the very same reasons, we previously failed to detect a difference in the plasma concentration of IL-26 between patients with moderate and severe COVID-19—or a significant correlation with length of hospital stay—in a small cohort (n = 49) that we used in a recent pilot study (18).

Second, we found that the plasma concentration of IL-26 is higher in male patients with COVID-19 than in female patients. Notably, both the severity and mortality of COVID-19 are known to be higher in men than in women (26, 27), and this difference has been attributed to a number of genetic, hormonal, and physiological variations (28). Given that we also see an increase in systemic IL-26 in patients with severe/critical COVID-19, our results motivate further research into potential associations between increased IL-26 and patient mortality.

Third, we show that the plasma concentration of IL-26 is higher in COVID-19 patients with comorbid obstructive lung disease (i.e., asthma and/or COPD), which agrees with previous studies on asthma (29–31). Although we are not aware of corresponding studies on systemic IL-26 in COPD, our current findings are compatible with the fact that the concentration of IL-26 is enhanced in the airways of patients with COPD (32). Nevertheless, in our study we have focused on systemic alterations in IL-26 during acute COVID-19 and it is possible that the inflammatory profile in the airways of these patients might look different. In addition, even though we did not detect any differences in the plasma levels of IL-26 between patients with COVID-19 who received corticosteroids (a common treatment for COVID-19 and asthma) and those who did not, we cannot exclude the possibility that other drugs taken during sample collection might have had an impact on the systemic levels of IL-26 in certain patients.

Fourth, we have previously shown that stimulation of TLRs involved in the innate recognition of SARS-CoV-2 triggers the release of IL-26 in several immune and structural cell types *in vitro* (3, 4, 16, 17), and we now report positive correlations between the plasma concentrations of IL-26 and two SARS-CoV-2 antigens *in vivo*. Nevertheless, our multivariable analysis does not support a direct association between having high systemic concentrations of SARS-CoV-2 antigens (above median) and having a plasma concentration of IL-26 above the LLOD. These seemingly contradictory findings suggest that although SARS-CoV-2 particles might be able to induce the production of IL-26 directly in the airways, the association between SARS-CoV-2 antigens and IL-26 production might be indirect at the systemic level. Importantly, SARS-CoV-2 particles can pass from the airways and into the bloodstream (viremia) where they trigger a potent and harmful hyperinflammatory response (33–37). In agreement with this, we found that patients with severe/critical COVID-19 had higher plasma levels of SARS-CoV-2 antigens. Thus, it seems likely that enhanced IL-26 production at the systemic level occurs because of the hyperinflammation associated with SARS-CoV-2 viremia and not because of direct SARS-CoV-2 recognition by pattern-recognition receptors (PRRs).

Fifth, it is known that virus-infected cells produce vast amounts of interferons, and we now report positive correlations between the plasma concentration of IL-26 and those of IFN-α2a, IFN-β, and IFN-γ. Notably, IFNs-α/β can modulate the expression of IFN-γ, while the genes of IFN-γ and IL-26 are located on the same chromosome, share an enhancer element that drives their expression, and are often co-expressed in different lymphocyte subsets (8–10, 38). In agreement with this, our multivariable analysis further supports the association between IL-26 and IFN-γ at the systemic level. Therefore, IL-26 seems likely to play complementary antiviral functions in COVID-19.

Sixth, we have now identified a positive correlation between the systemic concentration of IL-26 and that of extracellular dsDNA. Although extracellular dsDNA in plasma is a non-specific marker of NET formation that can also derive from tissue damage-related cell necrosis, there is a tight relationship between increased NET formation and tissue-damaging hyperinflammation. For instance, we and others have previously shown that patients with acute COVID-19 display increased neutrophil activation and NET formation, all of which correlates with harmful hyperinflammation in these patients (18, 39). Moreover, IL-26 is a neutrophil-mobilizing cytokine that can also bind and enhance the pro-inflammatory potential of extracellular DNA (4, 40, 41), and we have previously reported in a small pilot study that the plasma concentration of IL-26 correlates with several markers of hyperinflammation and tissue damage such as IL-8, TNFα, and lactate dehydrogenase (LDH) in patients with acute COVID-19 (18). Taken together, our findings support the notion that increased systemic IL-26 might enhance the harmful pro-inflammatory potential of extracellular dsDNA regardless of its origin during acute COVID-19.

In summary, our novel results link enhanced systemic IL-26 to severity of disease, male sex, and respiratory comorbidity in acute COVID-19. These results also associate enhanced systemic IL-26 with viral load and the magnitude of the classic antiviral immune response, plus extracellular dsDNA—an important sign of excessive neutrophil activation and tissue-damaging hyperinflammation in COVID-19. In view of the published evidence on the antimicrobial and neutrophil-mobilizing properties of IL-26, together with our recent finding that systemic IL-26 associates with hyperinflammation and tissue damage in COVID-19 (18), our novel results strongly forward IL-26 as an active player in acute COVID-19. Thus, IL-26 bears both antiviral and pathogenic relevance in COVID-19 and, given its accessibility in blood, it may constitute a potential target for clinical diagnosis, monitoring and treatment of this dangerous disease.

## Data Availability

The original contributions presented in the study are included in the article/**Supplementary Material**. Further inquiries can be directed to the corresponding author.
