# Supplementary material for: Systemic increase in IL-26 is associated with severe COVID-19 and comorbid obstructive lung disease
Source: Front Immunol. 2024 Oct 4;15:1434186. doi: 10.3389/fimmu.2024.1434186 (PMC11486738; doi:10.3389/fimmu.2024.1434186)
Supplement: Supplementary file 1 [file DataSheet1.docx]

Supplementary Material

1. Supplementary Figures

**Supplementary Figure 1. Relationship between the plasma concentration of IL-26 and the time between onset of symptoms and sample collection in patients with COVID-19.** Percentage of samples in which the plasma concentration of IL-26 fell below (black) or above (red) the lower limit of detection (LLOD) in each study group.

**Supplementary Figure 2. Relationship between the plasma concentrations of IL-26 and hypertension or diabetes type 2 in patients with COVID-19.** Comparisons of the plasma concentrations of IL-26 between patients with and without comorbid **(A)** hypertension or **(B)** diabetes type 2 including all patients with COVID-19 (n=178) by two-tailed unpaired Mann-Whitney test. Red horizontal lines represent the median. ns = not significant.

**Supplementary Figure 3. Relationship between the plasma concentrations of IL-26 and corticosteroid use in patients with COVID-19.** **(A)** Comparison of the plasma concentrations of IL-26 between patients with and without corticosteroid use including all patients with COVID-19 (n=178) by two-tailed unpaired Mann-Whitney test. Red horizontal lines represent the median. ns = not significant. **(B)** Percentage of samples in which the plasma concentration of IL-26 fell below (black) or above (red) the lower limit of detection (LLOD) in each study group.

**Supplementary Figure 4. Receiver operating characteristic (ROC) curve of the logistic regression model.** This curve was generated for the model described in Table 2.
